# Providers’ Perceptions of Telehealth Barriers Among Homebound Adults in in a Home-Based Primary Care Practice

**DOI:** 10.1093/geroni/igab046.2060

**Published:** 2021-12-17

**Authors:** Kate Moody, Alex Kalicki, Peter Gliatto, Emily Franzosa, Katherine Ornstein

**Affiliations:** 1 Icahn School of Medicine at Mount Sinai, New York, New York, United States; 2 Icahn School of Medicine at Mount Sinai, Icahn School of Medicine at Mount Sinai, New York, United States

## Abstract

The COVID-19 pandemic resulted in a dramatic shift to video-based telehealth use in home-based primary care. We conducted an online 11-item survey exploring provider perceptions of patients’ experience with and barriers to telehealth in a large HBPC program in New York City. More than one-third (35%) of patients (mean age of 82.7; 46.6% with dementia; mean of 4 comorbidities/patient) engaged in first-time video-based telehealth encounters between April and June 2020. The majority (82%) required assistance from a family member and/or paid caregiver. Among patients who had not used telehealth, providers deemed 27% (n=153) “unable to interact over video” for reasons including cognitive or sensory ability. Fourteen percent lacked caregivers. Physicians were not knowledgeable about patients’ internet connectivity, ability to pay for cellular plans, and video-capable device access. These findings highlight the need for novel approaches to facilitating telehealth and systematic data collection before targeted interventions to increase video-based telehealth use.

